# Low vaccination rates and awareness status in patients with rheumatoid arthritis: a nationwide cross-sectional survey study

**DOI:** 10.1007/s00296-025-05870-y

**Published:** 2025-04-22

**Authors:** Ali Kirik, Nilay Şahin, Merve Baykul, Hatice Bodur, Tuba Güler, Remzi Çevik, Sevcan Uğur, Yunus Durmaz, Ali Yavuz Karahan, Gül Devrimsel, Nuran Öz, Mehmet Nur Kaya, Yeşim Çağlar, Mehmet Tuncay Duruöz, Kemal Nas

**Affiliations:** 1 Department of Internal Medicine, Balıkesir University Faculty of Medicine, Balıkesir, Türkiye; 2 Department of Physical Medicine and Rehabilitation, Balıkesir University Faculty of Medicine, Balıkesir, Türkiye; 3 Department of Physical Medicine and Rehabilitation, Division of Rheumatology and Immunology, Sakarya University Faculty of Medicine, Sakarya, Türkiye; 4 Department of Physical Medicine and Rehabilitation, Yıldırım Beyazıt University Faculty of Medicine, Ankara, Türkiye; 5 Department of Physical Medicine and Rehabilitation, Ankara City Hospital, University of Health Sciences Ankara, Ankara, Türkiye; 6 Department of Physical Medicine and Rehabilitation, Faculty of Medicine, Dicle University, Diyarbakir, Türkiye; 7 Department of Rheumatology, Faculty of Medicine, Alanya Alaaddin Keykubat University, Antalya, Türkiye; 8 Department of Physical Medicine and Rehabilitation, Division of Rheumatology, Karabuk Training and Research Hospital, Karabuk, Türkiye; 9 Department of Physical Medicine and Rehabilitation, Faculty of Medicine, Uşak University, Uşak, Türkiye; 10 Department of Physical Medicine and Rehabilitation, Faculty of Medicine, Recep Tayyip Erdoğan University, Rize, Türkiye; 11 Department of Physical Medicine and Rehabilitation, Division of Rheumatology, Marmara University School of Medicine, Istanbul, Türkiye; 12 Department of Rheumatology, Hakkari State Hospital, Hakkari, Türkiye; 13 Department of Infectious Disease and Clinical Microbiology, Balıkesir University Faculty of Medicine, Balıkesir, Türkiye

**Keywords:** Rheumatoid arthritis, Vaccination, Haemophilus influenza vaccine, Pneumococcal vaccine, Surveys and questionnaires, Hepatitis vaccine

## Abstract

To examine the pneumococcal, haemophilus influenza, hepatitis A virus (HAV), and hepatitis B virus (HBV) vaccine rates and and predictors of vaccination among a Türkiye population of rheumatoid arthritis (RA) patients. In this multicenter, cross-sectional study, vaccination levels and related factors were questioned by face-to-face survey method during routine clinical examination of patients with RA followed in different regions of Türkiye. All statistical analyses were performed using the Statistical Package for the Social Sciences (SPSS) software package, version 26. A total of 715 patients (mean age 53.1 ± 13 years), of whom 552 (77.2%) were women, were included in this study. The rate of vaccine awareness education in the whole patient group was 40.1%. The vaccination rates for influenza, pneumococcal, HAV, and HBV were 34.3%, 21.8%, 12.3%, and 28.5%, respectively. It was noteworthy that the level of vaccine awareness education was a positive predictive factor affecting vaccination in all vaccine types (*p* < 0.001, for all vaccine types). The rates of low income were higher in the vaccinated group for pneumococcal (*p* < 0.001) and haemophilus influenza (*p* = 0.011) vaccine than in the unvaccinated group. The participants with living in a rural area were significantly higher in the unvaccinated group for HAV and HBV vaccine than in the vaccinated group (*p* < 0.001). The frequency of less than two medical visits per year was significantly higher in the unvaccinated group for pneumococcal (*p* < 0.014) and haemophilus influenza (*p* < 0.001 ) and HBV vaccine (*p* = 0.009) than in the vaccinated group. multivariate analysis found that rural area (for Haemophilus influenza and HAV vaccines) and financial income (Pneumococcal vaccine) in specifically were associated with increased vaccination rates (*p* < 0.001). Additionally, the rate of vaccination education was significantly higher in the vaccinated group for all vaccine types, which is noteworthy as a result of the positive effect of education on vaccination (*p* < 0.001). Vaccination awareness education and rates are low in primary prevention for RA patients. Vaccination awareness training is important in increasing the level of vaccine rates.

**Supplementary Information:**

The online version contains supplementary material available at 10.1007/s00296-025-05870-y.

## Introduction

Rheumatoid arthritis (RA) is the most common chronic inflammatory connective tissue disease worldwide [1]. Autoimmunity and synovial inflammation play a role in the pathogenesis of the disease, which is caused by genetic and environmental factors. While joint disease is the initial focus, over time, systemic inflammation and secondary clinical pathologies may occur [2]. In addition, the main treatment for RA patients is immunosuppressive drugs, and patients often have to survive with a suppressed immune system [3]. Inflammatory rheumatic diseases such as RA, along with musculoskeletal and other systemic symptoms, can lead to significant increases in morbidity and mortality [4].

Today, there is an increase in both community-acquired and hospital-acquired infections in RA patients. Although the reasons for this are complex, two important factors have been observed to play a role. These are autoimmunity, which plays a role in the pathogenesis of the disease, and immunosuppressive drugs such as corticosteroids, Disease-modifying antirheumatic drugs (DMARDs), and biologics, which are used to treat the disease along with systemic inflammation [5, 6]. In light of all this data, patients with RA are at increased risk of infection due to disease activity, immune dysfunction, and use of immunosuppressive medications [7, 8]. Therefore, vaccination is an important strategy to prevent widespread infections and their complications, as is the case in vulnerable populations with autoimmune diseases [9]. Additionally, the importance of vaccination is increasing day by day due to the increased risk of infection in RA patients. The main reason for this is that vaccine-preventable infectious diseases (pneumococcal, haemophilus influenza, viral hepatitis, herpes zoster, etc.) are frequently observed in the RA clinic, and vaccination against these diseases is effective [10, 11].

International guidelines recommend routine administration of haemophilus influenza, pneumococcal, and viral hepatitis vaccines in the clinical follow-up of RA patients [12, 13]. On the other hand, it has been reported in the current literature that vaccination rates in RA patients are not at adequate levels [14, 15]. Furthermore, to the best our knowledge, there is no national multicenter study investigating the vaccination level and associated factors in patients with RA in Türkiye. Based on this literature information, in this multicenter study, we aimed to examine the pneumococcal, haemophilus influenza, hepatitis A virus (HAV), and hepatitis B virus (HBV) vaccination rates of RA patients followed up in Türkiye and the variables affecting this rate.

## Materials and methods

### Study design and patient population

This study was designed as a multicenter, cross-sectional survey study from different geographical and cultural regions of Türkiye. All patients who were 18 years of age or older, had been diagnosed with RA for at least one year, and presented to our outpatient clinics were included in the study. RA diagnosis was classified with reference to 2010 ACR/EULAR criteria: joint symptoms; serology (including RF and/or ACPA); symptom duration, whether < 6 weeks or > 6 weeks; and acute-phase reactants (CRP and/or ESR) [16]. Exclusion criteria were pregnancy, immunodeficiency, bariatric surgery, specific causes of chronic liver disease, the history of renal replacement therapy, congestive heart failure, splenectomy history, dementia and malignancy. Patients were given detailed information about the questionnaire before the study, and informed consent was obtained from them. Local ethics committee approval was obtained for this study, and the study was designed in accordance with the Declaration of Helsinki.

### Questionnaire

At the beginning of this study, a team of relevant experts organized a draft questionnaire for patients in light of national and international guidelines for a detailed examination of vaccination. A literature review was conducted regarding vaccination prior to this survey. In the second stage, an information note was prepared in the initial part of a questionnaire regarding the possible benefits and risks. The questionnaire draft was designed to have a total of 24 questions under 7 main headings (Appendix 1). The majority of the questions were designed as single and multiple-choice answers. Questions with single answers presented as 3 or more Likert scales (e.g., yes/no/do not know) [17].

Firstly, age, gender, education, marital status, employment status, social insurance, smoking, income level, frequency of physician visits and place of residence were questioned. Secondly, medical history, medication use, vaccination history and vaccination status were questioned. Thirdly, the questionnaire draft was applied as a pre-test in the clinical research study group and necessary revisions were made in the light of the feedback. Lastly, the questionnaire was administered to a group of 30 target respondents. A round of revision was conducted for the survey draft. The draft of the survey was arranged in the current format after a revision and the average survey completion time was determined as 15 min.

The questionnaire was administered to the patients by face-to-face communication in all the centers where the study was conducted. Data obtained from patients were recorded in a highly secure manner within the framework of the Personal Data Protection Law and kept in an appropriate digital environment.

### Vaccination

In this study, patients were asked whether they had received the key pneumococcal, haemophilus influenza, HBV, and HCV vaccines recommended for routine RA patient care. Patients were also asked if they had received any clinical training in immunization and, if so, by whom.

### Statistical analysis

Written informed consent was obtained from study participants. All statistical analyses were performed using the Statistical Package for the Social Sciences (SPSS) software package, version 26. Normally distributed continuous variables are expressed as mean ± standard deviation (SD), skewed variables as median (interquartile range-IQR) and categorical variables are expressed as numbers (n) and percentages (%). The chi-square test was used in the analysis of qualitative independent data, and the Fischer exact test was used when the conditions for the chi-square test could not be met. A forward stepwise multivariate regression model was developed, using factors that had significant associations with Haemophilus influenza, Pneumococcal, HAV and HBV vaccine in the univariate analysis. Normally distributed data were compared using Student’s t-test. Statistical significance was defined as *p* < 0.05.

## Results

**Fig. 1:**
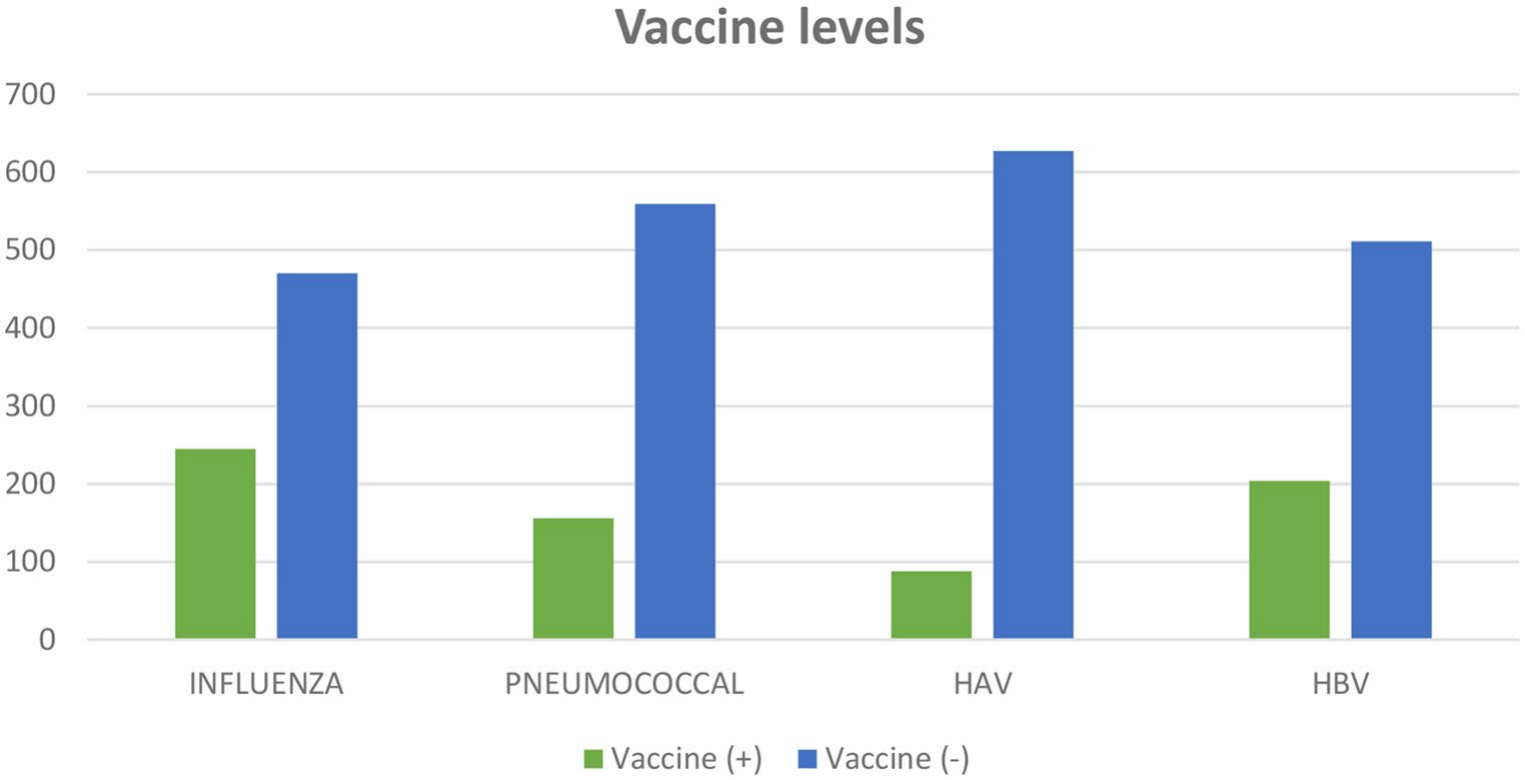
Vaccine levels of study group

**Fig. 2:**
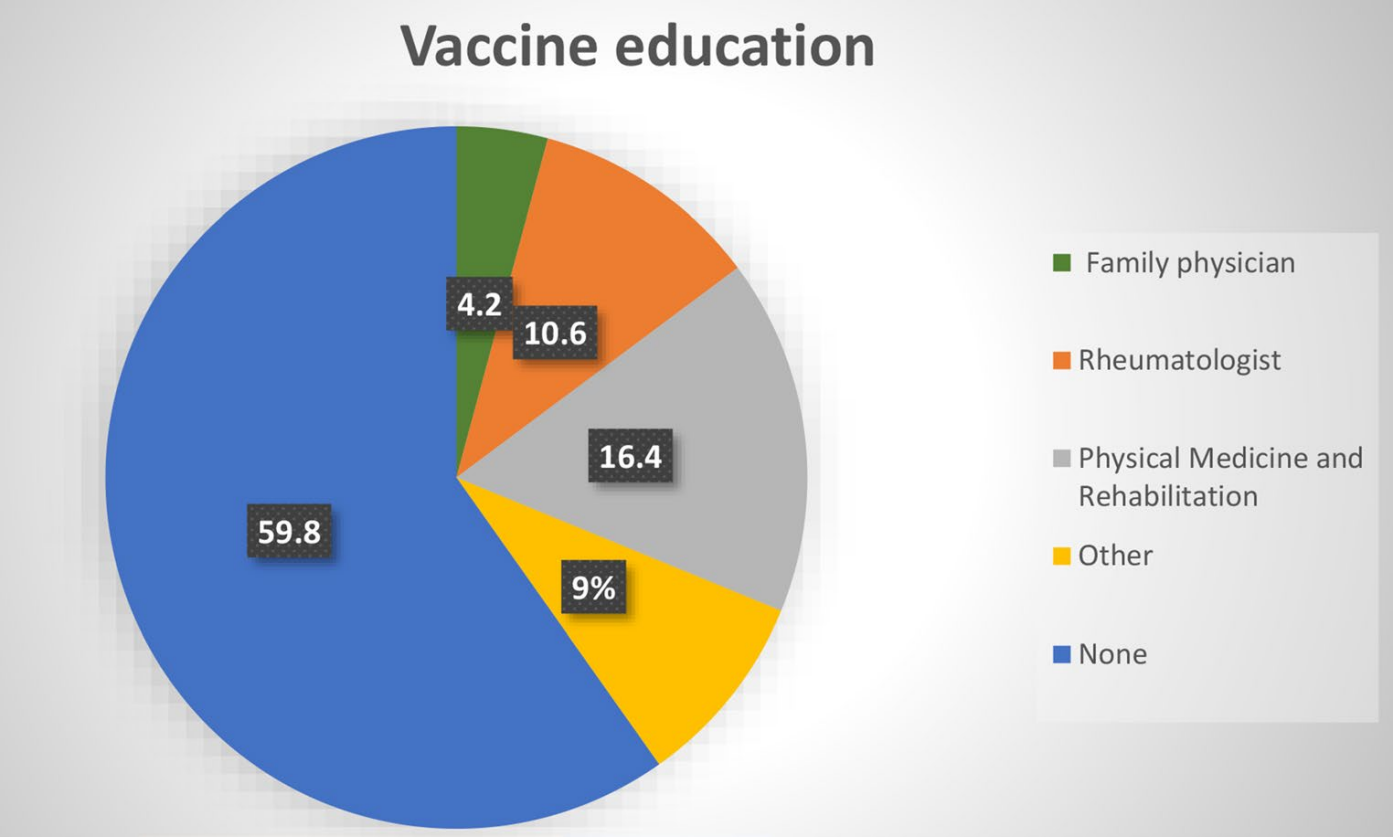
Vaccine education according to clinicians

A total of 715 patients (mean age 53.1 ± 13 years), of whom 552 (77.2%) were women, were evaluated in this study. The rate of vaccination awareness education in the whole patient group was 40.1%. It was observed that vaccination education was provided mostly by rheumatology (10.6%) and physical medicine (16.4%) and rehabilitation clinical departments (Figs. 1 and 2). Descriptive statistics of the patients are presented in Table 1.

**Table 1:** Baseline characteristics of study group

Variables	RA patients	%
***Sociodemographic***		
Mean age (years)	53.1 ± 13.1	n.a.
Female, n	552	77.2
High school education, n	225	31.5
Married, n	591	82.7
Working, n	172	24.3
Social insurance, n	674	94.3
Low financial income, n	499	69.8
Rural area, n	582	81.4
***Clinical***		
Smoker, n	150	21.0
Disease duration (years)	9.6 ± 7.9	n.a.
Frequency of visits, n		
Twice a year or more	126	17.6
Less than twice a year	589	82.4
***Comorbidity***		
DM, n	95	13.3
HT, n	193	27
*Medication*		
DMARD, n	591	82.7
Glucocorticoids, n	285	39.9
Immunomodulator, n	17	2.4
Biologics, n	175	24.5
***Vaccine education***		
Family physician, n	30	4.2
Rheumatologist, n	76	10.6
Physical Medicine and Rehabilitation, n	117	16.4
Other, n	65	9.0
None, n	428	59.8
***Vaccine levels***		
Influenza, n	245	34.3
Pneumococcal, n	156	21.8
HAV, n	88	12.3
HBV, n	204	28.5

Means are given with standard deviation (SD)*RA* rheumatoid arthritis, *DM* diabetes mellitus, *HT* hypertension, *DMARD* disease-modifying antirheumatic drug, *HAV* hepatitis A virus, *HBV* hepatitis B virus

### Haemophilus influenza vaccine

The proportion of patients who received influenza vaccine (*n* = 245) was 34.3%. The mean age was significantly higher in the vaccinated group (55.6 ± 13.5) compared to the unvaccinated group (51.8 ± 12.7) (*p* < 0.001). The rates of low income, vaccination education (*p* < 0.001 for all), and duration of disease follow-up (*p* = 0.048) were significantly higher in the vaccinated group compared to the unvaccinated group (Table 2). On the other hand, the rate of rural area and the frequency of less than two medical visits per year were higher in the non-vaccinated group (*p* < 0.001 for all). The logistic regression analysis reveals that the variables rural area (OR 8.21 95% CI: 5.11–13.17) and vaccine education (OR 6.53 95% CI: 4.48–9.51) exhibit considerable predictive power in relation to the outcome variable Haemophilus influenza vaccine (*p* < 0.001).

**Table 2:** Comparisons of the demographics and clinical parameters for influenza and Pneumococcal vaccine groups

Variable	Influenza (+) (*n* = 245)	Influenza (-) (*n* = 470)	*p* value	Pneumococcal (+) (*n* = 156)	Pneumococcal (-) (*n* = 559)	*p* value
Mean age (years)^*^	55.6 ± 13.5	51.8 ± 12.7	**< 0.001**	55.4 ± 11.8	52.4 ± 13.3	**0.008**
Female, n (%)	188 (76.7)	364 (77.4)	0.732	127 (81.4)	425 (76)	0.166
High school education, n (%)	71 (29)	154 (32.8)	0.297	41 (26.3)	184 (32.9)	0.103
Married n (%)	206 (84.1)	385 (81.9)	0.875	138 (88.5)	453 (81)	0.078
Working n (%)	57 (23.3)	115 (24.5)	0.423	33 (21.2)	139 (24.9)	0.182
Social insurance n (%)	236 (96.3)	438 (93.2)	0.062	150 (96.2)	524 (93.7)	0.194
Low financial income n (%)	200 (81.6)	299 (63.6)	**< 0.001**	121 (77.6)	378 (67.6)	**0.011**
Rural area n (%)	143 (58.4)	439 (93.4)	**< 0.001**	121 (77.6)	461 (82.5)	0.188
Disease duration (years)^*^	10.5 (9.1)	9.2 (7.3)	**0.048**	9.5 ± 7.1	9.7 ± 8.2	0.880
Frequency of visits n (%)^**^	174 (71)	415 (88.3)	**< 0.001**	117 (75)	472 (84.4)	**0.014**
Vaccine education n (%)	174 (71)	115 (24.5)	**< 0.001**	148 (94.9)	141 (25.2)	**< 0.001**

Statistically significant values with a p-value < 0.05 are shown in italics

^*^Means are given with standard deviation (SD)

^**^Less than twice a year

### Pneumococcal vaccine

The proportion of patients who received pneumococcal vaccination (*n* = 156) was 21.8%. The mean age was significantly higher in the vaccinated group (55.4 ± 11.8) compared to the unvaccinated group (52.4 ± 13.3) (*p* = 0.008). The rates of low income (*p* = 0.011) and vaccination education (*p* < 0.001) were higher in the vaccinated group than in the unvaccinated group (Table 2). However, the frequency of less than two medical visits per year was significantly higher in the unvaccinated group (*p* < 0.014). The logistic regression analysis reveals that the variables low financial income (OR 6.94 95% CI: 4.43–10.86) and vaccine education (OR 9.53 95% CI: 6.11–14.87) exhibit considerable predictive power in relation to the outcome variable Pneumococcal vaccine (*p* < 0.001).

### HAV vaccine

The proportion of HAV vaccinated patients (*n* = 88) was 12.3%. There was no significant difference in age between the vaccinated (53.9 ± 12.5) and unvaccinated (52.9 ± 13.2) groups (*p* = 0.504). The rate of vaccination education (*p* < 0.001) and the duration of disease follow-up (*p* = 0.008) were significantly higher in the vaccinated group (Table 3). On the other hand, being employed (*p* = 0.039) and living in a rural area (*p* < 0.001) were significantly higher in the unvaccinated group. The logistic regression analysis reveals that the variables rural area (OR 9.38 95% CI: 4.96–17.76) and vaccine education (OR 5.39 95% CI: 2.94–9.89) exhibit considerable predictive power in relation to the outcome variable HAV vaccine (*p* < 0.001).

**Table 3:** Comparisons of the demographics and clinical parameters for HAV and HBV vaccine groups

Variable	HAV (+) (*n* = 88)	HAV (-) (*n* = 627)	*p* value	HBV (+) (*n* = 204)	HBV (-) (*n* = 511)	*p* value
Mean age (years)^*^	53.9 ± 12.5	52.9 ± 13.2	0.504	54.7 ± 12.9	52.4 ± 13.1	**0.037**
Female, n (%)	72 (81.8)	480 (76.6)	0.271	160 (78.4)	392 (76.7)	0.704
High school education, n (%)	23 (26.1)	202 (32.2)	0.251	57 (27.9)	168 (32.9)	0.192
Married n (%)	76 (86.4)	515 (82.1)	0.379	171 (83.8)	420 (82.2)	0.607
Working n (%)	15 (17)	157 (25)	**0.039**	49 (24)	123 (24.1)	0.767
Social insurance n (%)	79 (89.8)	595 (94.1)	0.131	195 (95.6)	479 (93.7)	0.337
Low financial income n (%)	66 (75)	433 (69.1)	0.237	163 (79.9)	336 (65.8)	**< 0.001**
Rural area n (%)	41 (46.6)	541 (86.3)	**< 0.001**	146 (71.6)	436 (85.3)	**< 0.001**
Disease duration (years)^*^	11.1 ± 4.9	9.4 (8.2)	**0.008**	8.1 (6.7)	10.2 (8.3)	**< 0.001**
Frequency of visits n (%)^**^	68 (77.3)	521 (83.1)	0.222	155 (71.6)	434 (84.9)	**0.009**
Vaccine education n (%)	63 (71.6)	226 (36)	**< 0.001**	186 (91.2)	103 (20.2)	**< 0.001**

Statistically significant values with a p-value < 0.05 are shown in italics.,

^*^Means are given with standard deviation (SD)

^**^Less than twice a year

*HAV* hepatitis A virus, *HBV* hepatitis B virus

### HBV vaccine

The proportion of patients who received HBV vaccine (*n* = 204) was 28.5%. The mean age was significantly higher in the vaccinated group (54.7 ± 12.9) compared to the unvaccinated group (52.4 ± 13.1) (*p* = 0.037). The rates of low-income and vaccination education were significantly higher in the vaccinated group compared to the unvaccinated group (*p* < 0.001). However, living in a rural area (*p* < 0.001), having less than two medical visits per year (*p* = 0.009), and having a long disease follow-up period (*p* < 0.001) were significantly higher in the unvaccinated group (Table 3). The logistic regression analysis reveals that the variables vaccine education exhibit considerable predictive power in relation to the outcome variable HBV vaccine (OR 11.76 95% CI: 7.97–17.37, *p* < 0.001).

## Discussion

Our aim in this study is to increase vaccination rates in rheumatic diseases such as RA and to draw attention to the need in this regard. In recent years, especially in developed countries, there has been a reluctance to get vaccinated all over the world due to skepticism, hesitation and disbelief in the benefits and protection of vaccines. Rheumatologists can inform their patients about the safety and efficacy of vaccines in inflammatory rheumatic diseases and emphasize the importance of the vaccination process in reducing the risk of infection and complications. They can play an important role in reducing vaccine hesitancy and increasing vaccination rates in this group of patients by engaging in patient education initiatives and actively recommending and encouraging vaccinations. In the light of these data, the vaccination rates in the entire patient group was observed to be below the expected level. At the same time, only 40.2% of patients received vaccination education. However, older age (for influenza, pneumococcus, and HBV), urban residence (for influenza, HAV, and HBV), and high frequency of physician visits (for influenza, pneumococcus, and HBV) were determinants of multiple vaccinations. Interestingly, low income (for influenza, pneumococcal, and HBV) was observed at a higher rate in groups with higher vaccination rates. On the other hand, multivariate analysis found that rural area (for Haemophilus influenza and HAV vaccines) and financial income (Pneumococcal vaccine) in specifically were associated with increased vaccination rates. Additionally, the rate of vaccination education was significantly higher in the vaccinated group for all vaccine types, which is noteworthy as a result of the positive effect of education on vaccination.

### Haemophilus influenza vaccine

Currently, a high incidence of influenza-induced infectious diseases is observed in RA patients compared to the normal population. This situation increases the importance of influenza vaccination in the follow-up of RA patients [18]. On the other hand, the current literature emphasizes that influenza vaccination rates are not at an adequate level [19]. In a study by Hmamouchi et al. in which 3920 RA patients from 17 countries were evaluated, the mean influenza vaccination rate was 25.3%. In addition, the vaccination rate varied between countries from 1 to 66%. Furthermore, in countries where vaccination is more common, predictive factors for vaccination were found to be older age, lower disease activity, higher education level, use of biologic agents, absence of corticosteroid treatment, and presence of comorbidities [20]. In another study conducted by Ta et al. in Canada, the vaccination rate of RA patients before diagnosis was 38%, and this rate increased by only 8% after diagnosis to reach 46%. This shows that although the disease is diagnosed, the awareness and practice of vaccination are at a low level [21]. In fact, in the present study, the influenza vaccination rate was 34.3%, which is below the social vaccination target [22]. In addition, advanced age, vaccination education, urban residence, long disease follow-up, and frequent medical check-ups were significantly higher in the vaccinated group, which supports the current literature [20, 23]. Interestingly, the level of low financial income was higher in the vaccinated group in our study, which differs from the classical paradigm, emphasizing that vaccination increases in parallel with income level in the current literature [24]. In conclusion, the variables underlying the variation in influenza vaccination between countries are at different levels in our study.

### Pneumococcal vaccine

In studies in the current literature, several factors influence the pneumococcal vaccination rate, and this rate varies between countries. In a study conducted by Schmedt et al. in a large German cohort of approximately 200,000 patients, the vaccination rate was 4.4% in all high-risk conditions, and when disease-based vaccination rates were examined, it was observed that the highest rate was observed in RA patients with a history of immunosuppressive drug use (11.5%). This study also found that the vaccination rate tended to increase with increasing age [25]. In addition, in the analysis of pneumococcal vaccination performed by Matthews I. et al. in a group of approximately 100,000 patients in the United Kingdom, it was found that 13.6% of all patients were vaccinated during the one-year follow-up period and 32.0% during the four-year follow-up period. In addition, this study found that the average time from diagnosis to vaccination was five months [26]. Considering all this information, the pneumococcal vaccination rate in the present study was 21.8%, which was found to be low in accordance with the literature. Additionally, older people who had received vaccination education and had a higher frequency of physician visits were observed at a higher rate in the vaccinated group. Futhermore, similar to influenza vaccination, low financial income levels were observed at a higher rate in the pneumococcal vaccination group, which was found to be different from the literature.

### HAV and HBV vaccine

Although HAV and HBV vaccination is important in chronic inflammatory diseases, there is limited information in the current literature on patients who have received these vaccines in RA patients. In a study conducted by Qendro T. et al. in the Canadian population, 136 RA patients were evaluated, and the HBV vaccination rate was 33.6%. In this study, physician recommendation was found to be the most effective predictor of positive vaccination [27]. Furthermore, in another study conducted by Krasselt M. et al. in Germany, the HAV and HBV vaccination rates in RA patients were 8.5% and 11.8%, respectively [14]. In the present study, HAV (12.3%) and HBV (28.5%) vaccination rates were low, which is consistent with the current literature. As expected, receiving vaccination education and living in urban areas were significantly higher in the vaccination group for both vaccine types. In addition, the frequency of physician visits was higher in the HBV vaccinated group than in the unvaccinated group. On the other hand, the duration of disease follow-up was higher in the vaccinated group for HAV but not for HBV. It is important to show that one variable may have different effects on different vaccine types. In addition, the low financial income level in HBV vaccination was higher in the vaccinated group, and this result is different from that of the literature [27].

### Strengths and limitations

To our knowledge, there is no multicenter awareness study designed on vaccination in RA patients in Türkiye in the current literature. The study presented here is the first national multicenter vaccination awareness study conducted in Türkiye in a large cohort of RA patients. The strength of our study is that it is the first multicenter study in RA patients conducted in regions with different geographical and cultural characteristics, and it evaluates four types of vaccines (pneumococcal, influenza, HBV, and HCV vaccines) together. Additionally, this study analyzed the vaccination rates of patients as well as their vaccine awareness levels and education status. However, this study has several limitations. First, our study is a cross-sectional survey study, and health system registration data of patients could not be accessed. Second, patient self-reported data were used in the study, which could not prevent possible patient misreporting. Third, although our study was designed as a multicenter study, the data obtained do not reflect the whole country. Finally, with the data obtained in this study, it was not possible to investigate the extent to which correct interventions with patients could influence the vaccination rate.

## Conclusion

In conclusion, the majority of RA patients have an increased risk of infectious diseases due to immune system dysfunction and the use of immunosuppressive drugs. Therefore, many international guidelines in the current literature emphasize the importance of vaccine protection for some of these infections in RA patients (we should ask whether they have had retrospective influenza, pneumococcal, and hepatitis). On the other hand, vaccination awareness education and vaccination rates are low in primary prevention for RA patients. Increasing vaccination education, especially for patients, and increasing the frequency of physician visits stand out as paradigms that will increase vaccination levels. In addition, prioritizing age-based approaches to vaccination is important to increase vaccination rates. Moreover, there is a need to determine the reasons for the skepticism, hesitation and reluctance to get vaccinated due to lack of belief in the benefits and protection of the vaccines recommended for rheumatic patients and to develop new and effective comprehensive strategies that will positively affect the use of vaccines in this group of patients with immune system disorders.

## Electronic supplementary material

Below is the link to the electronic supplementary material.

Supplementary Material 1

## Data Availability

Data are available on reasonable request. Data are available from corresponding author on reasonable request.

## Declarations

### Disclaimer status

The authors, declare that this manuscript is original, has not been published before and is not currently being considered for publication. No part of this manuscript, including the text and graphics, has been copied, published or reproduced in any journal or book.
