# Supplementary material for: Low vaccination rates and awareness status in patients with rheumatoid arthritis: a nationwide cross-sectional survey study
Source: Rheumatol Int. 2025 Apr 22;45(5):116. doi: 10.1007/s00296-025-05870-y (PMC12014830; doi:10.1007/s00296-025-05870-y)
Supplement: Supplementary file 1 — Supplementary Material 1 [file 296_2025_5870_MOESM1_ESM.docx]

| **1. Sociodemographic characteristics** | |  |
| --- | --- | --- |
| 1. Age and gender | …………………. years | …………………… |
| 1. Education (High school) | Yes ( ) No ( ) | Do not know ( ) |
| 1. Married | Yes ( ) No ( ) | Do not know ( ) |
| 1. Working | Yes ( ) No ( ) | Do not know ( ) |
| 1. Social insurance | Yes ( ) No ( ) | Do not know ( ) |
| 1. Low financial income | Yes ( ) No ( ) | Do not know ( ) |
| 1. Rural area | Yes ( ) No ( ) | Do not know ( ) |
| **2. Clinical characteristics** | |  |
| 1. Smoking | Never ( )  Former ( )  Smoker ( ) |  |
| 1. Disease duration (years) | …………………. years |  |
| 1. Frequency of visits | Twice a year or more ( )  Less than twice a year ( ) |  |
| **4. Comorbidity** | |  |
| 1. Diabetes Mellitus | Yes ( ) No ( ) |  |
| 1. Hypertension | Yes ( ) No ( ) |  |
| **5. Medication** | |  |
| 1. DMARD | Yes ( ) No ( ) |  |
| 1. Glucocorticoids | Yes ( ) No ( ) |  |
| 1. Immunomodulator | Yes ( ) No ( ) |  |
| 1. Biologics | Yes ( ) No ( ) |  |
| **6. Vaccine education** | |  |
| 1. Family physician | Yes ( ) No ( ) |  |
| 1. Rheumatologist | Yes ( ) No ( ) |  |
| 1. Physical Medicine and Rehabilitation | Yes ( ) No ( ) |  |
| 1. Other | Yes ( ) No ( ) |  |
| **7. Vaccine status** | |  |
| 1. Influenza | Yes ( ) No ( ) | Do not know ( ) |
| 1. Pneumococcal | Yes ( ) No ( ) | Do not know ( ) |
| 1. HAV | Yes ( ) No ( ) | Do not know ( ) |
| 1. HBV | Yes ( ) No ( ) | Do not know ( ) |
